# Notes

**Published:** 1898-06

**Authors:** 


### NOTES.

### LETTER ON SUCCESSFUL TREATMENT OF SARCOMA.

Thamesville, Ont., June 13, 1898.

*J. McFadden Gaston, Esq., M.D.,* 1½ *Edgewood ave., Atlanta, Ga.*

DEAR DOCTOR: I dare say you would care to know how we are progressing with Cataphoresis. I do not know, of course, how much credit is due to your line of treatment, but the fact is that my brother is at present in excellent health, with no appearance of disease about him, whereas over a year ago I had thought that further attempts to save his life would be useless, and I was backed up in this by several good men. Certainly had we allowed the case to take a natural course he would have been dead before now. The tumor was removed as often as it recurred—averaging once in two months. I commenced treatment by Cataphoresis on June 24, 1897, and have continued it since, together with the administration of succus alteraus and Donovan's solution. I had to remove tumor three times, however, viz.: August 30; October 21; December 31; and now for nearly six months have not had any sign of trouble. Before commencing this treatment the tumor had been removed five times with an average interval of two months.

MICROSCOPIC REPORTS.

First—Adeno Sarcoma of Testicle. Dr. Cavan, Toronto.

Second—Round Celled Sarcoma (first recurrence). Dr. Anderson, Toronto.

Third—Carcino Sarcoma (third recurrence). Dr. Anderson, Toronto.

Fourth—Adeno Carcinoma (fifth, sixth and seventh recurrences). Dr. Cullen, Johns Hopkins Hospital.

Of course it is too soon to speak with confidence, but perhaps this seeming prospect of success even, may encourage some one else to persevere.

My experience with this case would lead me in a similar one, to remove the recurring growths as early as possible. Seeing that there is no room for a radical operation, I would remove only enough tissue to insure complete removal of growth—not cutting as wide as possible, but saving tissues for future operations. I would avoid using ligatures if at all possible to control bleeding points without them, and I would persevere with Cataphoresis, using the positive electrode near the tumor and the negative at a considerable distance from the same. Twice in this case I have had to dissect very carefully the growth from the urethra and felt sure that a recurrence would take place there, but was agreeably disappointed to find the next appearance at a different part, thus getting as good a result as if I had cut wider, and with a very great saving of discomfort to the patient. The last removal of the growth did not extend much more than one-fourth inch away from the tumor.

I have often wondered whether that patient with disease of penis, whom you mentioned in one of your letters, had received benefit or not. You said another medical man had asked you about your treatment and proposed trying it with him.

I have to thank you again for your kind assistance and courtesy in this case, and only hope that the termination of it may be such as will place me in a position to more publicly acknowledge the same. I remain very truly yours,

R. N. FRASER.

---

Dr. F. S. Bourns, Professor of Pathology and Bacteriology, has been granted a leave of absence of one year. He has been appointed on Gen. Merritt's staff to the position of surgeon with the rank of major to serve in the Phillipine expedition. Dr. Bourns' experience admirably qualifies him for the part assigned, and his advice is sought by the government departments in many matters of importance concerning our new possessions in the East. He spent four years of wandering among those islands and in Borneo in the interest of the Minnesota Scientific Society, for the purpose of making advanced study of the birds of the Phillipines. He is familiar with the topography of the country, and speaks many of the island dialects. Such an experience, which falls to the lot of a few, will be of great value when applied to furthering the interest of the government.

In the absence of Dr. Bourns, Dr. Claude A. Smith, who has been associated with him, will have charge of his affairs and is prepared to make pathological examinations of urine, faeces, sputum, etc., and for which they have fitted out offices in the Grand building.

---

Dr. W. Owens, Southern Medical College, class of '97, has returned from a year's stay abroad and has taken offices in the English-American Building. He expects to limit his practice to diseases of the eye, ear, throat and nose.

---

Dr. Marion Hull, formerly of Athens, is now associated with Dr. J. Scott Todd.

